# Autocrine and Paracrine Effects of Vascular Endothelial Cells Promote Cutaneous Wound Healing

**DOI:** 10.1155/2021/6695663

**Published:** 2021-04-10

**Authors:** Yang Lu, Yuhao Yang, Liling Xiao, Shenghong Li, Xuan Liao, Hongwei Liu

**Affiliations:** ^1^Department of Plastic Surgery, The First Affiliated Hospital of Jinan University, Key Laboratory of Regenerative Medicine, Ministry of Education, Guangzhou, Guangdong Province 510630, China; ^2^Department of Orthopedics, The First Affiliated Hospital of Jinan University, No. 613, Whampoa Avenue West, Guangzhou, Guangdong Province, China

## Abstract

**Background:**

When vascular endothelial cells are subjected to external stimuli, paracrine hormones and cytokines act on adjacent cells. The regulation of the biological behaviour of cells is closely related to the maintenance of organ function and the occurrence and development of disease. However, it is unclear whether vascular endothelial cells affect the biological behaviour of cells involved in wound repair through autocrine and paracrine mechanisms and ultimately play a role in wound healing. We aimed to verify the effect of the autocrine and paracrine functions of vascular endothelial cells on wound healing.

**Materials and Methods:**

ELISA was used to detect platelet-derived growth factor, basic fibroblast growth factor, epidermal growth factor, and vascular endothelial growth factor in human umbilical vascular endothelial cell-conditioned medium (HUVEC-CM). Different concentrations of HUVEC-CM were used to treat different stem cells. CCK-8 and scratch assays were used to detect the proliferation and migration ability of each cell. A full-thickness dorsal skin defect model was established in mice, and skin wound healing was observed after the local injection of HUVEC-CM, endothelial cell medium (ECM), or normal saline. H&E staining and immunofluorescence were used to observe the gross morphology of the wound tissue, the epithelial cell migration distance, and the expression of CD3 and CD31.

**Results:**

HUVEC-CM promotes the proliferation and migration of epidermal stem cells, skin fibroblasts, bone marrow mesenchymal stem cells, and HUVECs themselves. Furthermore, HUVEC-CM can promote angiogenesis in mouse skin wounds and granulation tissue formation and can accelerate wound surface epithelialization and collagen synthesis, thereby promoting wound healing.

**Conclusion:**

Our results clearly suggest that it is practicable and effective to promote wound healing with cytokines secreted by vascular endothelial cells in a mouse model.

## 1. Introduction

Skin is a complex organ containing different cell types, which are composed of epidermal, dermal, and hypodermal tissues along with various other components, such as vasculature, sensory neurons, the skin immune system, and other appendages [1]. The epidermis serves as a physical barrier for the skin. It can limit harmful ultraviolet radiation through pigmentation, as well as provide sensory nerve conduction, immune surveillance, and temperature regulation. The dermis is the main component of the skin, mainly comprising a complex extracellular matrix, cells, blood and lymphatic vessels, nerves, and ducts. Between the epidermis and the dermis is the basement membrane, which is an extracellular matrix structure. Below the dermis is a layer of subcutaneous fat tissue (also known as the hypodermis), which provides temperature regulation, and tissue contouring [2, 3]. In the process of wound healing, endothelial cells, keratinocytes, and fibroblasts are the main types of cells involved in reepithelialization and granulation tissue formation. Therefore, continuous and mutual communication between epidermal and dermal cells plays a key role in skin development, homeostasis, and repair [4]. Our skin is perpetually exposed to various detrimental physical or chemical factors. Failure to repair damaged skin in a timely manner could lead to dehydration, infection, or even death. Therefore, rapid wound healing is essential. However, certain wounds are very challenging to treat in clinical work, especially those related to lesions and blood vessel obstructions. Angiogenesis is an important part of the wound healing process and is the basis for tissue repair. Vascular endothelial cells are the principal cells of blood vessels and are critical for the formation of new blood vessels [5, 6]. The proliferation, migration, and sprouting of vascular endothelial cells are important for the creation of new blood vessels, which is one of the most vital functions of vascular endothelial cells [7]. It is very important that in angiogenesis, oxygen, nutrition, and bioactive substances are provided for wound repair. Therefore, the role of vascular endothelial cells in wound healing is extremely important.

The results of the current study indicate that vascular endothelial cells form a very large secretory gland that extends to include the surface of the entire vascular tree, with an area of approximately 400 m^2^ [8]. Endothelial cells in various organs are affected by external stimulation by paracrine hormones and cytokines. This stimulation regulates the biological behaviour of adjacent smooth muscle cells, monocytes, macrophages, fibroblasts, and organ-specific cells, as well as organs [9]. Research on angiogenesis and wound healing has received extensive attention. However, few people have explored the relationship between vascular endothelial cell secretions and wound healing [10].

**Figure 1:**
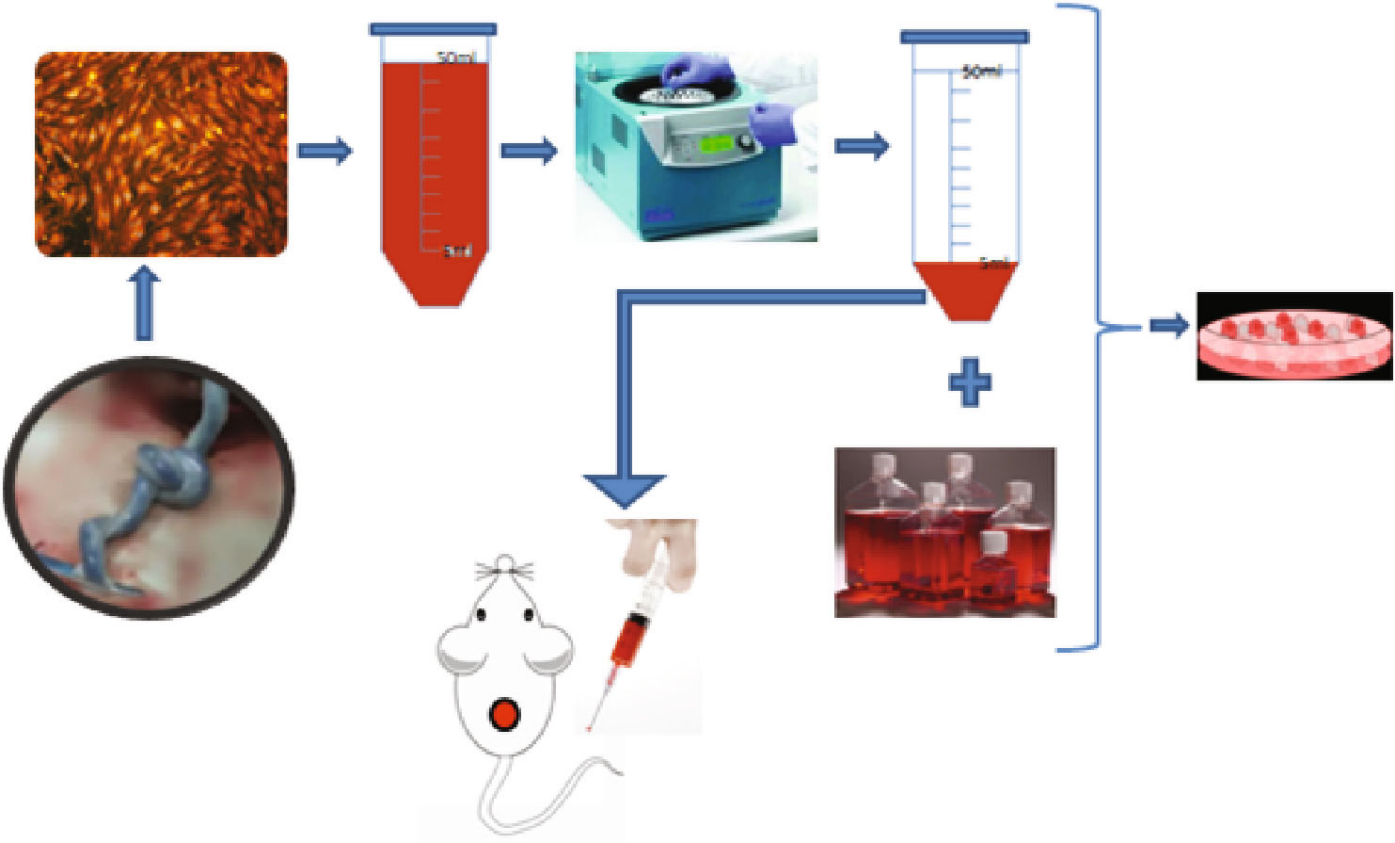
Flow diagram: the supernatant of P3 umbilical vein endothelial cells was collected, filtered, and concentrated tenfold using vacuum freeze-drying centrifugation. One part was mixed with several factors that participate in wound repair, and the other part was used to treat full-thickness skin defects in mice.

We determined the therapeutic efficacy of human umbilical vascular endothelial cell- (HUVEC-) conditioned medium (HUVEC-CM) because angiogenic properties are considered to be an important step in regeneration and HUVECs are a representative endothelial cell population. The concise flow diagram of our research is shown in Figure 1. First, using cytokine expression assays, we observed that concentrated HUVEC-CM contains several cytokines, such as platelet-derived growth factor (PDGF), basic fibroblast growth factor (bFGF), epidermal growth factor (EGF), and vascular endothelial growth factor (VEGF), which are known to be important for normal angiogenesis and wound healing. Then, we found that the HUVEC secretions promoted the proliferation and migration of epidermal stem cells (ESCs), skin fibroblasts (Fbs), bone marrow-derived mesenchymal stem cells (BMSCs), and HUVECs themselves. Moreover, the application of secreted factors obtained from concentrated HUVEC-CM to cutaneous defects accelerated wound healing, tissue granulation, reepithelialization, and collagen synthesis compared with the application of vehicle control medium and normal saline. These data provide a theoretical basis for understanding the relationship between the secretions of vascular endothelial cells and the healing of skin wounds and can provide additional methods for promoting wound healing and treating various diseases associated with vascular dysfunction.

## 2. Material and Methods

### 2.1. Isolation, Culture, and Identification of HUVECs

Maternal umbilical cord (15-20 cm) was collected, soaked in 0.25% chlorhexidine for 10 minutes, and then washed with sterile saline 3 times; the haematoma was cut where clamp marks were observed. The lumen was rinsed, and the umbilical vein was rinsed with sterile saline. The lumen was filled with 0.1% collagenase I and digested in an incubator at 37°C in 5% CO_2_ for 10-15 minutes. The umbilical cord was then removed, and the collagenase solution was collected. The umbilical cord was washed with endothelial cell medium (ECM), which was collected. The cells were collected by centrifugation at 1000 rpm for 5 minutes and were then cultured in ECM+10% foetal bovine serum (FBS, Gibco) and maintained at 37°C in 5% (*v*/*v*) CO_2_. Upon reaching 70-80% confluence after 7-9 days, adherent cells were isolated using trypsin and passaged 4 times for use in experiments to detect the expression of CD31 and von Willebrand factor (vWF) [11].

### 2.2. Preparation of Concentrated HUVEC-CM and Analysis of Cytokine Expression

HUVECs were cultured to passage 3. Upon the cells reaching approximately 80% to 90% confluence, serum-free cell culture medium was added. After 24 hours, the supernatant of the HUVEC culture was aspirated and centrifuged for 5 minutes at 1000 r/min; the supernatant was then collected and frozen at -20°C. The frozen endothelial cell supernatant was placed in a vacuum freeze-drying centrifuge and freeze-dried for 20 hours to obtain a frozen sample of 10-fold concentrated supernatant. Cytokines are released by HUVECs. The levels of PDGF, bFGF, EGF, and VEGF were measured using ELISA kits (R&D Systems, Minneapolis, MN) [12].

### 2.3. Isolation, Culture, and Identification of hESCs, Fbs, and hBMSCs

Normal human foreskin was collected. After subcutaneous fat and loose connective tissue were removed, the foreskin was cut into pieces 1 cm × 1 cm in size and digested. The epidermis was separated and digested to obtain hESCs and skin Fbs. The hESCs were resuspended in prepared keratinocyte serum-free medium (K-SFM, LONZA) and inoculated in culture flasks prepared with human type IV collagen [13]. The Fbs were cultured in Dulbecco's modified Eagle medium (DMEM)+10% foetal bovine serum (FBS) and maintained at 37°C in 5% (*v*/*v*) CO_2_ at passage 2. Passage 2 hESCs and passage 3 Fbs were used in the experiments. Fresh bone marrow free of haematological malignancy was added to an equal volume of 10% FBS in DMEM [14]. After centrifugation at 1200 r/min for 8 minutes, the pellet was collected. hBMSCs were obtained by density gradient separation. The cells were cultured in DMEM+10% FBS and placed at 37°C in 5% (*v*/*v*) CO_2_ at passage 2 [15].

### 2.4. Proliferation and Wound-Healing Assay In Vitro

The concentrated HUVEC-CM was mixed with the complete medium for each cell type to prepare 5 different gradient concentrations of conditioned medium (0%, 33%, 50%, 66%, and 100%). The different media were applied to HUVECs, hESCs, Fbs, and hBMSCs. The CCK-8 kit (Sigma) was used to measure cell proliferation and the resulting absorbance values. The scratch test was used to test cell migration [16]. ImageJ software was used to measure the remaining area of the wound.

### 2.5. Excisional Wounding, Grouping, and Topical Treatment

All protocols were approved by the Institutional Animal Care and Use Committee of Jinan University, China. All animal procedures were performed in strict accordance with the recommendations in the Guide for the Care and Use of Laboratory Animals produced by the National Institutes of Health. The experimental animals consisted of thirty male C57 mice (18-22 g) obtained from the Sun Yat-sen University Animal Center. After an intraperitoneal injection of phenobarbital sodium was administered for anaesthesia, the back hair was shaved and the skin was disinfected with 75% alcohol. A full layer of skin was cut from the back of the mice using a round 8 mm hole punch. The back muscle layer was exposed, and the wound surface was allowed to heal naturally without sutures or dressings [17]. These mice (*n* = 30) were randomly divided into 3 groups: The HUVEC-CM experimental group, in which animals received an injection of HUVEC-CM around the wound daily at a fixed time from the 1st day; the ECM group, in which pure ECM was injected around the wound daily at a fixed time from the 1st day; and the blank control group, in which normal saline was injected around the wound daily at a fixed time from the 1st day. The wound closure status was evaluated using a digital camera after 0, 1, 3, 7, and 11 days of treatment. A full-thickness skin sample from the wound site and surrounding normal tissue was collected using a circular 10 mm punch centred on the wound surface. The tissue was immersed in 4% paraformaldehyde and fixed at 4°C.

### 2.6. Analysis of the Wound Area

The area of the wound was calculated using ImageJ software. The following formula was used to calculate the wound area: area (%) = (wound size/original wound size) × 100%.

### 2.7. Histological Evaluation

Tissue sections were stained with haematoxylin and eosin (H&E) for light microscopy [18]. The stained sections were analysed using ImageJ software by tracing the areas of wound epithelialization and granulation tissue formation. Masson staining was performed to detect collagen fibres [19]; the intensity of blue staining corresponds to the amount of collagen deposition.

### 2.8. Immunofluorescence Staining

Paraffin-embedded sections first underwent standard deparaffinization and rehydration procedures and were then probed with fluorescein isothiocyanate-anti-CD3 antibody (ab135372; Abcam) and anti-CD31 antibody (ab9498; Abcam) overnight at 4°C [20]. Next, the sections were incubated with horseradish peroxidase-conjugated secondary antibodies. The nuclei were then counterstained with 4′,6-diamidino-2-phenylindole dihydrochloride [21].

### 2.9. Statistical Analysis

Values are expressed as the mean ± SD in the text and figures. The data were analysed by one-way ANOVA (SPSS 19.0 for Windows). Differences between two groups were evaluated for statistical significance using Student's *t*-test. Values of *P* < 0.05 were considered significant in all analyses.

## 3. Results

### 3.1. Cytokines Released by HUVECs

**Figure 2:**
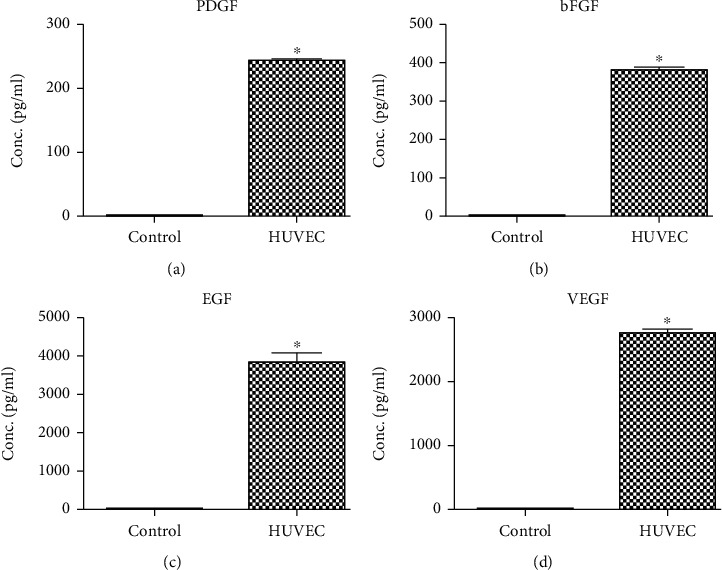
Cytokine analysis of HUVEC-CM using ELISA. HUVEC-CM contains PDGF, bFGF, EGF, and VEGF. HUVEC, hESC, Fb, and hBMSC proliferation and migration in vitro after treatment with various concentrations of HUVEC-CM.

Analysis of the HUVEC-CM revealed that the HUVECs expressed significant amounts of several growth factors, including PDGF (243.47 ± 8.57 pg/ml), bFGF (382.04 ± 13.64 pg/ml), EGF (3,823 ± 429 pg/ml), and VEGF (2,762 ± 74.7 pg/ml). Furthermore, the serum-free ECM did not contain PDGF, bFGF, EGF, or VEGF. These results indicate that the HUVECs secreted key factors for wound healing (^∗^*P* < 0.05) (Figure 2).

### 3.2. Proliferation and Migration of HUVECs, hESCs, Fbs, and hBMSCs

**Figure 3:**
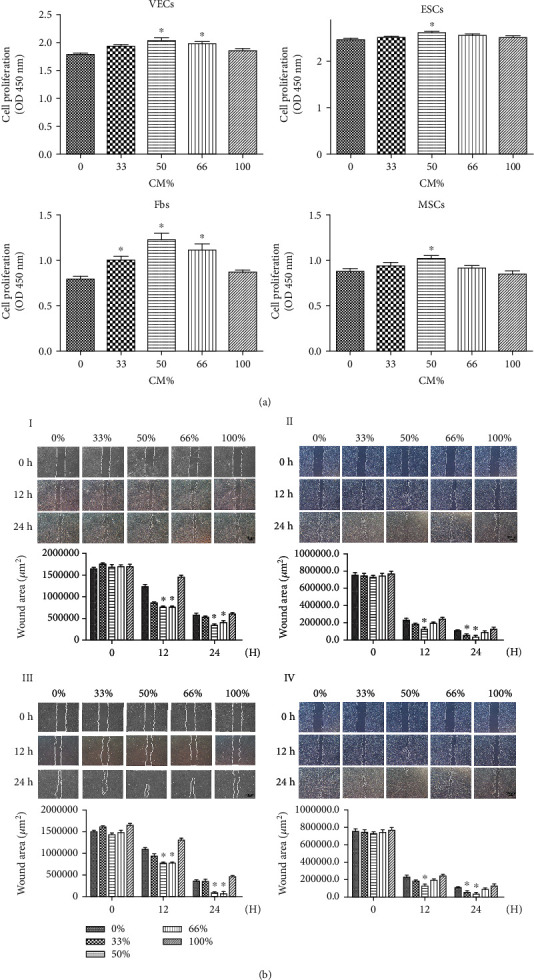
(a) Measurement and analysis of the proliferation of HUVECs (I), hESCs (II), Fbs (III), and hBMSCs (IV) using five concentrations of HUVEC-CM. (b) Microscope view of the measurement and analysis of the migration of HUVECs (I), hESCs (II), Fbs (III), and hBMSCs (IV) using five concentrations of HUVEC-CM.

The results showed (Figure 3) that the addition of HUVEC-CM to the culture medium of hESCs promoted the proliferation and migration of HUVECs, hESCs, Fbs, and hBMSCs. HUVECs, hESCs, Fbs, and hBMSCs showed the greatest capacity for proliferation and migration at a HUVEC-CM concentration of 50% (^∗^*P* < 0.05).

### 3.3. Wound Area and Days to Epithelialization

**Figure 4:**
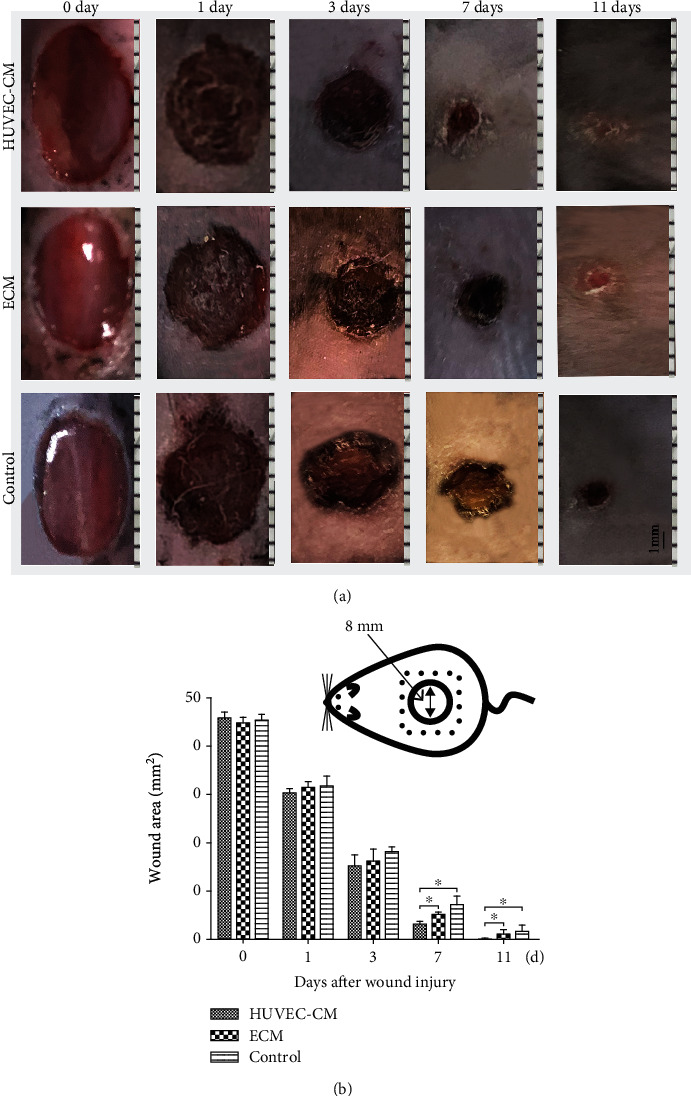
Macroscopic lesion appearance. (a) A standardized wound area (a circular, 8 mm diameter, full-thickness skin defect) on a mouse. (b) Measurement and analysis of the wound area. Histological assay.

Figure 4(a) shows that the wound healing rate was faster in the HUVEC-CM group than in the pure ECM group and the blank control group. From the third day to the eleventh day, compared with the other two groups, the experimental group showed a significantly reduced wound area and an accelerated healing rate. Animals in the HUVEC-CM group showed complete healing by the 11th day, but those in the ECM group and the blank control group still showed bleeding. On the 7th and 11th days after wounding (Figure 4(b)), the healing rate in the HUVEC-CM group was significantly faster than that in the other two groups (*P* < 0.05). On the 7th day, the wound area in the HUVEC-CM group was approximately 27%, while the wound area in the ECM group was approximately 35% and that in the control group was approximately 45%; the differences were statistically significant (*P* < 0.05).

### 3.4. H&E Staining

**Figure 5:**
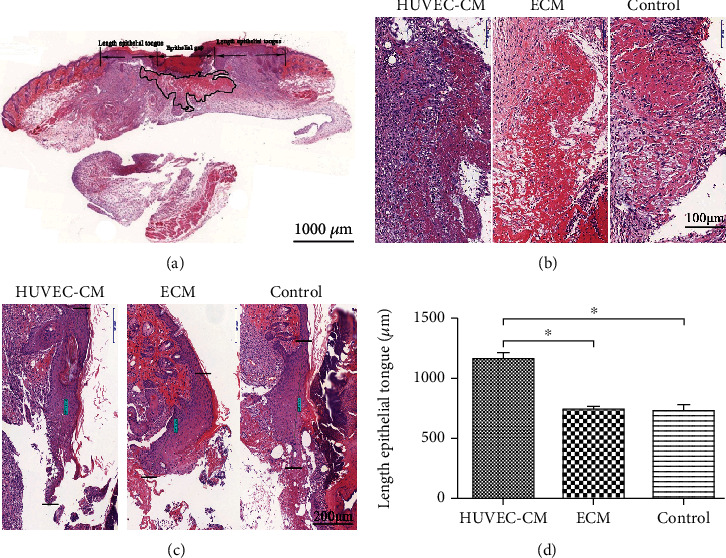
Histological analysis of the skin after H&E staining. (a) A schematic of a transverse section is depicted for proper identification of the respective regions. (b, c) Images of granulation tissue were obtained from the healing wound. Three days after wounding, the HUVEC-CM group showed greater inflammatory cell infiltration than did the other two groups. The epithelial cell migration distance seven days after wounding is the distance between the black lines. (d) Measurement and analysis of the length of new epithelial tissue (*P* < 0.05).

The mouse skin wound sections were subjected to histological examination (Figure 5(a)). The distance between the black lines represents the epithelial cell migration distance. The black curve outlines fresh granulation tissue (scale, 1000 *μ*m). Histological examination showed fresh granulation tissue on day 3 after wounding. Compared with the other two groups, the HUVEC-CM group showed greater inflammatory cell infiltration under the wound (scale, 100 *μ*m). On day 7 after wounding (Figures 5(b) and 5(c)), the distance of the cells from the marginal origin of the epithelium was 1161.5 ± 97.4 *μ*m in the HUVEC-CM group, 738.6 ± 43.6 *μ*m in the ECM group, and 731.3 ± 79.3 *μ*m in the blank control group (scale, 200 *μ*m). The reepithelialization in the HUVEC-CM group was greater than that in the other two groups (*P* < 0.05) (Figure 5(d)).

### 3.5. Masson Staining

**Figure 6:**
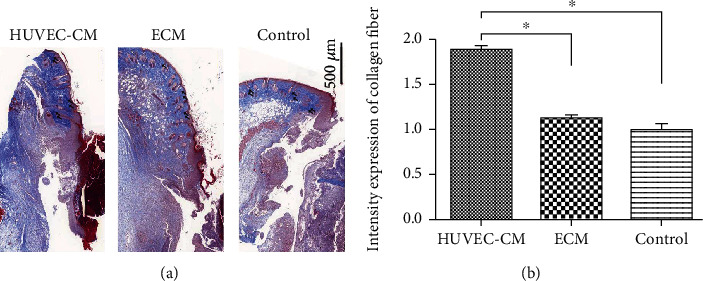
Masson-stained sections showing blue staining for collagen. (a) Seven days after wounding, collagen in the HUVEC-CM group was denser than that in the other two groups (arrows). (b) Measurement and analysis of the intensity of collagen deposition.

The Masson-stained sections showed blue-stained collagen (Figure 6). On the 7th day, collagen staining in the HUVEC-CM group was denser than that in the control group and the blank group. Collagen fibres were whirlpool-shaped, and the collagen was thick and dark. Collagen in the ECM group and the blank control group was slender. As the collagen gap increased, the staining became lighter, and the collagen was observed to be arranged in a reticular structure (scale, 500 *μ*m) (*P* < 0.05).

### 3.6. CD3 and CD31 Expression Levels

**Figure 7:**
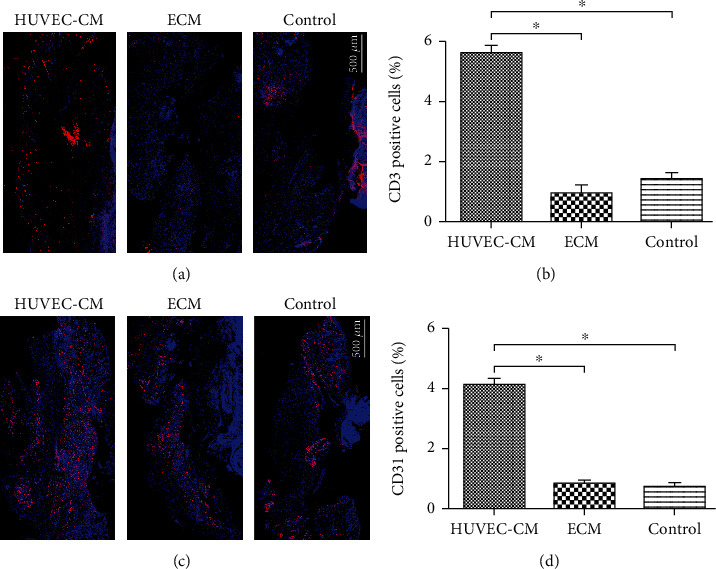
Expression of lymphocyte marker CD3 and angiogenesis marker CD31 at the defect site. (a) Immunofluorescence microscopy showing endothelial cell immunostaining for CD31 (red) and measurement and analysis of CD31-positive cells. (b) Immunofluorescence microscopy showing endothelial cell immunostaining for CD3 (red) and measurement and analysis of CD3-positive cells.

On day 3 after wounding (Figure 7(a)), CD3 (red) immunofluorescence was observed at the edge of the skin. The expression of lymphoid cells in the HUVEC-CM group was greater than that in the other two groups (scale, 500 *μ*m) (*P* < 0.05). This indicated that lymphocyte chemotaxis in the wound tissue was greater in the HUVEC-CM group than in the other two groups. On day 7 after wounding (Figure 7(b)), CD31 (red) immunofluorescence was observed at the edge of the skin. The HUVEC-CM group showed significantly more CD31 expression in epidermal basal cells and underlying soft tissues than did the other two groups (scale, 500 *μ*m) (*P* < 0.05), indicating greater angiogenesis at the defect site in the HUVEC-CM group than in the other two groups.

## 4. Discussion

Certain wounds are difficult to treat clinically, especially those associated with vascular lesions and obstructions. This suggests that vascular endothelial cells are not only important for their role in constituting blood vessels but may also regulate various cells in wound repair by organizing reconstruction through autocrine and paracrine mechanisms. An increasing numbers of studies have revealed that vascular endothelial cells constitute an inert blood container and can be considered a large “endocrine organ” [10]. In our study, we found that HUVECs, which are representative of the endothelial cell population, can condition culture medium with factors that promote the proliferation and migration of hESCs, skin Fbs, and hBMSCs.

Vascular endothelial cells undergo angiogenesis through proliferation, migration, and budding and play a key role in wound healing. After skin tissue is damaged, granulation tissue is formed during the healing process. Angiogenesis leads to the formation of blood vessels, which are important components of granulation tissue that provide oxygen, nutrients, and bioactive substances to the wound site and thus play a vital role in wound healing [2]. During skin healing, endothelial cells germinate from existing capillaries to the wound and grow new blood vessels to provide nutrition for wound healing. In addition, endothelial cells mediate the chemotaxis of inflammatory cells from the blood vessels to the wounded skin tissue, and these inflammatory cells can secrete inflammatory factors and extracellular matrix to promote the proliferation of basal cells [22–24].

hESCs are present in the basal part of the epidermis and proliferate and differentiate to replace outer, terminally differentiated cells, further renewing the tissue structure [25, 26]. The death and shedding of the superficial layer of cells and the division and proliferation of basal ESCs maintain a balance [27]. To maintain the normal tissue structure and preserve the stability of the intracellular environment, the epidermis is maintained through lifelong self-renewal [28]. Therefore, the mechanism by which ESCs proliferate and migrate is of great importance in promoting the complete repair of functional and structural skin damage.

Fbs play a pivotal role in the repair of wounds through the proliferation of cells and the formation of the extracellular matrix [29]. On the one hand, Fbs proliferate massively during the early stage after trauma and extensively synthesize and secrete collagen fibres and matrix components, which, together with new granulation tissue and capillaries, fill wounds to cover the wound surface [30]. On the other hand, during the later stage after trauma, Fbs secrete a large amount of collagenases that act in tissue repair and reconstruction [31].

BMSCs can differentiate into osteoblasts, chondrocytes, adipocytes, myocytes, and nerve cells under appropriate conditions [32, 33]. BMSCs also secrete CCL2 for efficient delivery to the injured spinal cord and recruit macrophages to drive their conversion toward a neuroprotective phenotype, which also plays a key role in preventing the degeneration of motor neurons [34]. Studies have shown that BMSCs are also present in the peripheral blood. During the inflammatory phase of wound healing, BMSCs are recruited to the wound and undergo proliferation and differentiation into a variety of cells that function in wound repair, including keratinocytes, endothelial cells, pericytes, and mononuclear cells. BMSCs also secrete a large number of growth factors and cytokines to promote wound healing [35].

The above cells all play critical roles in wound repair. Our results demonstrate that culture medium conditioned by vascular endothelial cells can promote the proliferation and migration of various cells involved in wound healing in vitro. This shows that vascular endothelial cells can accelerate the proliferation and migration of themselves as well as ESCs, skin Fbs, and BMSCs through the secretion of autocrine and paracrine factors to promote wound healing.

However, wound healing involves a variety of cells and multiple links. First, inflammatory cells migrate to the wound. Then, keratinocytes, Fbs, and endothelial cells migrate and proliferate, leading to reepithelialization, neovascularization, and granulation tissue formation. This is accompanied by tissue reconstruction, including maturation of the extracellular matrix. Ultimately, the structure and function of the system is restored. Vascular endothelial cells may affect other important aspects that can in turn promote wound healing [36]. To explore the mechanisms of the autocrine and paracrine functions and effects of vascular endothelial cells in wound healing, we applied HUVEC-CM to wounds, thus overcoming the problems associated with using cells. The findings of our study in a mouse skin defect model show that HUVEC-CM can promote angiogenesis and granulation tissue formation as well as accelerate wound surface epithelialization and collagen synthesis, thus promoting wound healing.

## Data Availability

The data used to support the findings of this study are available from the corresponding author upon request.
